# Bartonella Endocarditis: A Missed Diagnosis in Medical Practice

**DOI:** 10.7759/cureus.19309

**Published:** 2021-11-06

**Authors:** Yixin Zhang, Jorge Verdecia, Okechukwu Mgbemena, Malleswari Ravi, Michael Sands

**Affiliations:** 1 Internal Medicine, University of Florida College of Medicine – Jacksonville, Jacksonville, USA; 2 Infectious Disease, University of Florida College of Medicine – Jacksonville, Jacksonville, USA; 3 Cardiology, University of Florida College of Medicine – Jacksonville, Jacksonville, USA

**Keywords:** blood culture-negative endocarditis, immunofluorescence assays, mobile aortic vegetation, aortic valve insufficiency, bartonella endocarditis

## Abstract

Here, we present the case of a 52-year-old patient who presented with fever, chills, and weight loss. Further workup revealed *Bartonella* endocarditis of the aortic valve. After six weeks of antibiotics, a follow-up transthoracic echocardiogram showed a decrease in the size of vegetation. Serologic testing based on epidemiologic history should be obtained for the workup of blood culture-negative endocarditis.

## Introduction

Blood culture-negative endocarditis (BCNE) continues to be an elusive diagnosis due to the absence of positive blood culture and high likelihood of normal findings on echocardiogram; therefore, it is challenging to meet the modified DUKE criteria [1]. BCNE caused by *Bartonella* species first gained attention in 1993 in immunocompromised and immunocompetent individuals [1]. Since then, more than 40 *Bartonella* species have been discovered, with *B. henselae* and *B. quintana* reported in most BCNE cases [1,2]. Because of their slow-growing nature and poorly stainable properties, detection of antibodies in the host is needed to confirm the etiology [1]. Our case emphasized the importance of early clinical suspicion and a diagnostic approach for *Bartonella* endocarditis to start treatment early with the appropriate antibiotic regimen.

## Case presentation

**Figure 1:**
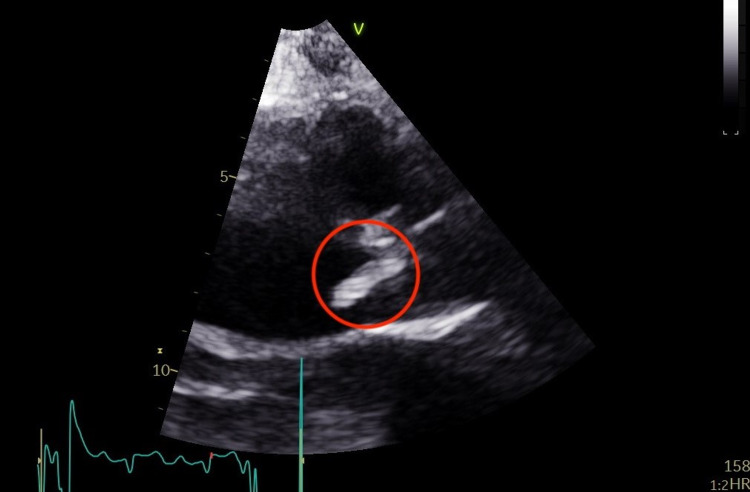
Large mobile vegetation (indicated by the red circle) measuring 2.1 × 0.6 cm attached to the right coronary cusp of the aortic valve with surrounding tissue destruction freely prolapsing into the left ventricle outflow tract.

**Figure 2:**
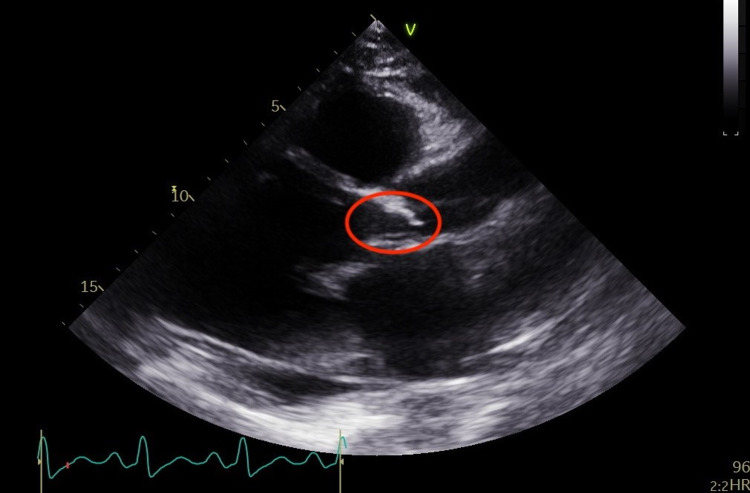
Treatment-responsive aortic vegetation (red circle) now measuring 0.7 × 0.3 cm with surrounding fibrosis.

A 52-year-old male presented to the emergency room complaining of intermittent fever, chills, weight loss, and night sweats for the past six months. His medical history was significant for human immunodeficiency virus (HIV)/acquired immunodeficiency syndrome (AIDS) (CD4 count of 54 cells/mL but adherent to antiretroviral therapy), remote history of intravenous drug use (last use one year ago), and treated hepatitis C infection. He was homeless but currently living in a group home. At presentation, there was no leukocytosis, but he was febrile at 102.1°F. Physical examination revealed decreased breath sounds with low oxygen saturation in the mid 80% requiring nasal cannula; however, no murmurs, Osler nodes, or Janeway lesions were noted. His labs were significant for an elevated C-reactive protein of 113.2 mg/L (0.00-5.00 mg/L) and N-terminal pro b-type natriuretic peptide level of 3,973 pg/mL (0-450 pg/mL). Chest computed tomography with contrast showed enlarged mediastinal lymph nodes and diffuse airspace disease within the right lung. Blood cultures collected before antibiotic administration remained negative at five days. His transthoracic echocardiography (TTE) and transesophageal echocardiogram (TEE) revealed a large, freely prolapsing vegetation on the right coronary cusp of the aortic valve, measuring 2.1 × 0.6 cm with associated severe aortic regurgitation (Figure 1). Infectious Disease was consulted seven days after admission due to negative blood cultures and echocardiogram findings. Serologic testing for *Bartonella* was obtained given the epidemiological history of homelessness, HIV/AIDS, and exposure to cats. Testing revealed a positive immunoglobulin G (IgG) titer of 1:2,560 for* B. henselae* and IgG titer of 1:640 for *B. quintana*. The patient completed an inpatient course of gentamicin for the first two weeks and doxycycline and rifabutin for six weeks. Cardiothoracic Surgery was consulted, but the patient was deemed high risk for surgical intervention. Repeat TTE at the end of the antibiotic therapy showed improved aortic regurgitation with a marked reduction in the size of the vegetation to 0.7 × 0.3 cm (Figure 2). The modified DUKE criteria were met given the echocardiogram, history of intravenous drug use, fever, and serology. Tuberculosis was ruled out with subsequent negative results on QuantiFERON, bronchoalveolar lavage acid-fast smear, *Mycobacterium tuberculosis* nucleic acid amplification, and culture. Due to the high risk of septic embolization, lack of early surgical intervention, and immunocompromised state, a decision was made to extend his antibiotic therapy for six more weeks.

## Discussion

*Bartonella* spp. are the second most common causative organism for BCNE after *Coxiella burnetii* [2]. *B. quintana* is the causative agent in approximately 75% of all cases, followed by *B. henselae* in approximately 25% of all infective endocarditis cases [1]. It was first identified by Alberto Barton, a Peruvian microbiologist, in the early 1900s [3]. These zoonotic species have different ways of transmitting the disease to humans, for example, through a bite or scratch. Vectors such as fleas, lice, ticks, and sand flies have been recognized to transmit different *Bartonella* spp. [1,2]. Of the human-relevant diseases, two are globally distributed, namely, *B. henselae*, responsible for cat scratch disease, and *B. quintana*, responsible for Trench fever [1,2]. These bacteria can infect multiple organs, including the skin, eyes, brain, liver, blood, and heart [4].

While it is known that *Bartonella* infection can remain in the erythrocytes for weeks to months, clinical manifestations depend on the immune status of the host [1]. The majority of patients present with non-specific symptoms, predominantly fever, fatigue, and weight loss [1,4]. Splenomegaly is an important physical finding seen in 40% of patients [1].

*Bartonella* is a gram-negative coccobacillus that is slow-growing, stains poorly, and requires heme to grow [1,2]. The sensitivity of blood cultures is only approximately 20%; even with valvular tissue culture, sensitivity remains less than 30% [1]. In most US laboratory facilities, bacterial cultures are discarded after five to seven days, which is problematic given that* Bartonella* spp. can take up to 21 days to grow [1]. Histopathological stains of the surgical tissue are neither sensitive nor specific [1,4-6]. In recent years, where molecular testing has become available, polymerase chain reaction (PCR) 16S RNA of the valvular tissue has shown a sensitivity and specificity of 70-98% [1,5,6]. However, microbial DNA can persist for months following infection, in which case a positive PCR does not imply ongoing infection [5]. Serologic testing such as immunofluorescence assays and enzyme-linked immunosorbent assays are the most useful tests for the diagnosis of *Bartonella* endocarditis, but they are not part of the DUKE criteria yet [1,4,5]. IgG titers of *B. henselae* of >1:64 with a four-fold titer rise at least two weeks apart have a sensitivity of 88-98% [1,2]. A titer of >1:800 is considered suggestive of endocarditis, but levels of <1:800 do not exclude diagnosis [1,2,4-7].

It is worth mentioning that *Bartonella* antibody titers may be positive in asymptomatic, healthy individuals but at a much lower concentration [2,7]. Cross-reactivity to IgG assays of other *Bartonella* spp., *Chlamydia*, and *Coxiella* is common, which reduces the specificity of the test [1,2,6,7]. Our patient’s IgG titers to *B. quintana* were also positive, most likely due to cross-reactivity from *B. henselae* infection. Delay in diagnosis and treatment is associated with adverse outcomes [8]. In approximately 50% of cases, cusp perforation, flail, or both is seen in aortic valve infective endocarditis [8]. Treatment has primarily been concentrated in two-drug combination therapy, with gentamicin as an addition or part of the regimen [1,4]. Duration of treatment ranges from four to six weeks without definitive guidelines [1].

## Conclusions

Here, we presented a case of BCNE caused by *B. henselae*. Bartonella endocarditis is usually indolent and presents with non-specific symptoms, requiring clinicians to have a high index of suspicion for early diagnosis and treatment. In patients who have predisposing risk factors (e.g., immunocompromised state, intravenous drug use, or exposure to endemic areas), it is essential to include appropriate serologic testing in the workup of BCNE and fever of unknown origin. It will be prudent that the subsequently updated guidelines include IgG titer as part of the modified DUKE criteria.
